# Editors’ Table

**Published:** 1857-09

**Authors:** 


### Editors' Table.

CURIOSITIES OF MINERAL SPRINGS. —One of the greatest natural curiosities, is the uniform temperature and mineral impregnation of the water of a spring during long periods of time. We have good evidence for believing that the temperature of certain thermal springs, known and resorted to by the inhabitants of ancient Rome, has undergone no change down to the present day.

A great contrast is often offered in the extreme proximity of cold and hot springs to each other. In the valley of the Nerbada, Northern Hindostan, a hot spring gushes out, at a distance of only five or six paces from a cold one; and at a pass through the Himalaya range, close to the hot springs of Nobea, near Le, small streams of cold water issue. The hot springs at the western part of the great plateau or table land of Thibet the highest in the world, after having been agitated and churned in the great granite reservoirs or basins in which they rise, flow over or escape through clefts, and unite to form a rivulet, which a little more than a mile distant from its source is entirely frozen over. Often the ebullition becomes so violent in the middle of certain reservoirs, that great columns of water rise and fall periodically, as if forced out by an immense pump. Over these springs dense vapors continually rise in the air, and are condensed in the form of white clouds. The whole scene reminds us forcibly of the Geysers of Iceland, which these springs of Thibet so closely resemble. In the Himalaya range a strong contrast is exhibited in an arch of snow forty feet thick, extending across the nascent hot stream of Jumna-tree the temperature of which at this elevation, 10,849 feet above, is 194° F., more than equivalent to the heat of boiling water at the common level of the ocean. These springs issue from a ledge of rock, only ten to twelve feet above the bed of the river. Above the ledge the hot water forces its way through a crevice, in a smoking jet of five or six feet in height, which has melted the snow around to a distance of from twenty to thirty yards. The rills of hot water which issue from the laminæ of rocks above referred to, when united, form a considerable stream. The Hindoos bathe in a pool of the water of the river, in which rises a hot spring of considerable size, so that they obtain a bath of nature's tempering, nearly warm.

Heat and cold are in close embrace in the "valley of hot waters" in the Chilian Andes, where from under the peak of the *Cervade Azufre*, which forms part of an enormous mass of ore, issues a torrent of hot water, of the temperature of 134° F. From the centre of this valley of thermal springs, a stream pure as crystal flows out. On the summit of one of the spurs of the Andes, to the north of St. Jago, the capital of Chili, two springs rise, one hot, the other cold, the streams of which are united by a canal of only eighty feet in length, so as to form warm baths. At the Hot Springs in Virginia, the visitor can lay one hand on a cold and the other on a warm rill at the same moment. This is done with equal ease in the case of a cold rivulet, and a warm spring in one of the Feejee Islands. In the valley of Turners, in St. Michael's (one of the Azores), the two kinds of springs are so near each other, that the thumb may be placed in one of the temperature of 70° to 80° F., and the forefinger of the same hand in another, the heat of which is 190° to 200° F. At the Caldeeras, other hot springs in this island, in the crater of an extinct volcano, there is an oval pond, filled partly by hot water rising in the mud, and partly by means of hot and tepid and cold springs in other parts of the crater, the waters of which are collected into a smaller pond, and let into the larger one as occasion may require. When pond number one is filled for use, the contents resemble warm milk and water, wherein gas and steam are blowing constant bladders, which float lazily along the surface, and at length burst and form a creamy scum.

Not only are springs subterraneous, but they are also submarine. The main ones are seen bubbling up to the surface of the sea, and sometimes diffusing their heat to a considerable distance. Examples of this nature are met with in the island of Milo in the Levant, and at Vanua Levu, one of the Feejee Islands. In a bay of the latter stands a knoll of basalt, measuring about thirty feet by fifteen, which is covered with two feet of water at high tide. At the centre of this island-rock, which is partially hollowed out, there are several jets of boiling water. Similar phenomena are seen in the small islands in a bay (Braidenord) of the northwestern part of Iceland. Innumerable fresh-water and many mineral and thermal springs gush out from the beds of rivers and lakes, as the instance of the hot spring rising in the Sutledge, previously noticed.

The periodical rise and fall of certain springs constitutes another of the curiosities worthy of notice. This intermission is accompanied by the extrication of a large quantity of gas, which is usually carbonic acid. The evolution of gas or vapor goes on to a certain extent until it produces a strong pressure and consequent elevation of the water in the spring, which then falls until there is another accumulation of gas or of vapor. Intermitting springs are most common in volcanic districts; examples to this effect are furnished in the Geysers of Iceland, and in the hot springs of St. Michael and New Zealand. Colonel Fremont describes a spring in the region of the Great Salt Lake, in a volcanic district, which projects its water to the height of three feet from the ground, at irregular intervals. A subterranean noise is heard at the same time, which, together with the motion of the water, readily suggested its name of Steamboat Spring. Of a similar intermittent character are the Mineral Spring in Hungary, the saline one at Kissungen in Bavaria, and the one in Canada, forty miles from Montreal.

The Great Geyser, after many hours of tranquillity, projects its boiling water from a level of 12 or 14 feet below the surface of the ground to a height of 100, sometimes 150, feet above, the eruption being preceded by a thundering noise and shaking of the ground. The salt spring of Kissungen ebbs and flows five times daily, giving out enormous quantities of gas. In all these cases there is never an interruption to the supply of water in the spring, but only an increase of the quantity at certain intervals.

The most extraordinary example of intermission is that of the heat of a thermal spring, in a jungle between Banda and Bohuree, in Hindostan. Its usual temperature is 124° F.; but in the month of April, at the time of the full moon, this is reduced to 94° F., so as to allow of the natives, who assemble in large numbers for the purpose, bathing in its waters. Among the hydrographic curiosities of Cashmere is tbe ebbing and flowing spring at Sardi Breri, near the great gushing spring of Kolzen Nag. The spring is in the form of a small basin, eight feet deep, and three or four yards wide. About the time of the No Rez, or new day, as the vernal equinox is termed, a little more water than usual is observable in the basin; but it subsides again. Two months later, the water ebbs and flows rapidly for a quarter of an hour, three times a day, morning, noon, and evening. Hindoo faith attributes great virtues to the water at its rise on the 13th of June. The cause of the ebb and flow is supposed to depend on the different degrees of heat at different hours of the day, by which a greater or less quantity of snow is melted, and water furnished thereby to the spring.

---

Philadelphia Hospital.—Many of our readers are aware that this hospital, located at Blockley, on the west bank of the Schuylkill, is the great eleemosynary institution of this city, accommodating annually many thousand inmates affected with all kinds of diseases and injuries to which "flesh is heir." At this moment it contains nearly 2500 persons, most of them in need of professional aid. The number of deaths is from 500 to 800 a year. We mention these data to show what immense advantages the establishment *might* afford if it were properly conducted, as a theatre for clinical teaching and the study of pathological anatomy. As originally organized, it had its regular medical and surgical staff; and for many years it was the great clinical school of the country. Men of great distinction, such as Jackson, Dunglison, Gibson, Horner, Mitchell, Morton, Harlan, Pancoast, and Gerhard, were engaged in its service; and pupils flocked to it from all parts of the country to receive instruction in medicine, surgery, and midwifery. Many of the resident physicians — nearly always young graduates—have since risen to usefulness and distinction, dispensing the blessings which they received from the opportunities they derived from their sojourn in the establishment. By and by, the spirit of caprice seized the Trustees of the Hospital, and all of a sudden, without any provocation whatever on their part, the medical and surgical attendants were dismissed in a body, and clinical instruction suspended. Subsequently, through the influence, chiefly, of a few determined and influential physicians, the old order of things was, in some degree, reinstated, with the difference, however, that the Superintendent, instead of being a layman, as he had been originally, was now a medical man, with permission to exercise, virtually, all the rights and privileges of a medical and surgical chief. The medical and surgical staffs were, in fact, mere subordinates, subject to the will and caprice of the Superintendent. They had, it is true, the right to prescribe and to operate, as well as to deliver clinical instruction; it often happened that the most grievous obstacles were thrown into their way, and their functions were, therefore, frequently merely of a nominal character. The number of students, meanwhile, was comparatively small; for, although the two staffs comprised a great amount of the most able and efficient *materiel*, yet it was quite impossible, under the circumstances, to make the teaching as attractive and useful as it had been under the old *regime*. Still they were ready and willing to go on, inspired with a desire to do all they could for the benefit of the inmates of the establishment, the honor of the Philadelphia profession, and the good of medical science. The measure of their indignation was well-nigh full, when, two months ago, it completely overflowed, in consequence of the appointment to the office of Superintendent, of an individual, who of all men in the country, was probably the most obnoxious to the Philadelphia profession. We allude to Dr. James McClintock. This man, the founder of a medical school in this city, and for many years a teacher of anatomy and surgery, here and elsewhere, abandoned the profession about 1853, became, as is well-known, a public vendor of a number of secret medicines bearing his name, and largely advertised in the newspaper press of the day. It was in consequence of this step, that the American Medical Association, at their meeting at Detroit, in 1856, struck his name from the list of membership. Thus dishonored, first by his own act, and then by that of the most august medical tribunal of the country, Dr. McClintock was elected by the Guardians of the Poor of Philadelphia, who are all Trustees of the Philadelphia Hospital, to the office of Superintendent of one of the greatest charities in the world. The action of this body was, as might have been expected, immediately followed by the resignation of the medical and surgical staff, and of all the resident physicians save one. Dr. McClintock, we are still more sorry to say, was recommended for the post he now occupies, by one of the recently elected Vice-Presidents of the American Medical Association, by several Surgeons of the United States Navy, and by a number, happily, we believe, a small one, of practitioners of this city! We have at present no comments to offer respecting the conduct of these gentlemen, further than to express our deep regret that they should have so far forgotten what is due to their profession as to sign a testimonial in favor of Dr. McClintock as a candidate for so responsible and honorable a position. We are willing to believe that they were asleep when they affixed their sign-manual to the document, and that their personal feelings for an old friend got the better of their judgment. But, while we would throw over them the mantle of charity, we must express, in the most unequivocal terms, our unqualified disapprobation of their proceedings. The motto of every right-thinking medical man should be, "My profession first, my friend next." Of the conduct of the Trustees of the Hospital we have nothing to say; an irresponsible political body, who have no just conception of the honor and dignity of the profession, or of the requirements of medical science, what else could have been expected of them? If Esau could sell his birthright for a mess of pottage, there are men among them, medical men, too, we are told, who would not hesitate to sell the honor of the profession for an equally sensual consideration.

We have said more about this matter than we had at first intended. As public journalists, jealous of the honor and dignity of the profession, we have a duty to perform, and we should scorn ourselves if we could stand by, and see that profession wronged and abused in any manner, without expressing our sense of disapprobation at its aggressors. We will only add that the College of Physicians of Philadelphia, and the Philadelphia County Medical Society, whose resolutions we publish below, and whose honor has been so wantonly outraged in this transaction, should exert themselves in every possible way, to correct the evil here spoken of. We are satisfied that none but the most thorough and official measure will be of any avail. Are they ready to act in the matter?

At a meeting of the College of Physicians of Philadelphia, held July 1st, 1857, the following Preamble and Resolutions were unanimously adopted:

Whereas, The Board of Guardians of the Poor, at their meeting of the 8th of June last, elected to the office of Chief Resident Physician of the Philadelphia Hospital, Blockley, Dr. James McClintock, whose name had been stricken from the roll of the American Medical Association, at its annual session, held in the City of Detroit, in May, 1856, in consequence of his having abandoned the ranks of the Profession, and assumed the degrading position of a manufacturer and vendor of empirical and secret medicines; therefore be it

*Resolved*, That the selection of the said individual to fill the important and honorable office above referred to, made in the face of the verdict of the Association, a verdict by virtue of which he is no longer recognized as a member of the Medical Profession, is indignantly pronounced to be a gross and gratuitous outrage on the feelings of every member of that profession, not only in this city but in the country at large, and as such meets with the unqualified disapprobation of the College of Physicians.

*Resolved*, That the College takes this early opportunity of tendering its cordial and entire approbation of the course pursued by such of the Assistant Resident Physicians of the Institution mentioned above—namely, Drs. C. P. Tutt, of Va.; J. H. Berrien, of Ga.; J. Cummiskey, of Philadelphia; Thomas Marshall, of Va.; X. X. Xaupi, of Mo.; and J. S. Coleman, of Ga.—as have, at the sacrifice of most valuable opportunities of clinical instruction, promptly and unhesitatingly resigned the position they therein held, sooner than serve under a chief outlawed by the highest medical tribunal of the land, and with whom no physician, alive to a sense of self-respect, and having at heart the honor and dignity of his calling, can professionally associate. In so doing, the College further tenders to those gentlemen the assurance that, by thus acting, they have evinced a moral courage and an appreciation of the high obligations imposed upon them by the noble mission upon which they have recently entered worthy of all praise, and have thereby raised themselves in the estimation of every honorable member of the Medical Profession.

*Resolved*, That the College has learned, with unfeigned satisfaction, that the entire professional corps attached to the Institution alluded to, have, for the same reason, and to repel the insult offered to them by the recent action of the Board of Guardians, resigned their respective posts.

*Resolved*, That in the opinion of the College none of its members can justifiably accept office under or hold professional intercourse with the individual referred to in the above resolution, whether in the Institution so improperly placed under his control or elsewhere; and that by following a different course such members would become amenable to the censure of the College, and expose themselves to the penalties which the individual himself would have incurred had he been a member thereof.

*Resolved*, That the College would learn with deep regret and a feeling of shame, that any of its members had promoted, directly or indirectly, either by letter or otherwise, the election to a post of honor and emolument such as that of Physician in Chief of the largest Hospital in this State, of an individual who for the very grave offence referred to in the preamble has been cast from the folds of the profession, and cannot re-enter it without a formal reversal of the verdict under the effect of which he now labors; and that the said College would think itself obliged, did an event of the kind alluded to occur, to apply to the one guilty of so palpable an infraction of the Code of Ethics by which its members are governed, the penalties imposed on the abettors of quackery.

*Resolved*, That the College would learn with equal disapprobation that any of its members have recommended to others to take or retain office in an Institution whose Chief Physician, like the present incumbent in the Blockley Hospital, no longer holds an honorable position, if any, in the Medical Profession.

At a special meeting of the Philadelphia County Medical Society, held June 30th, 1857, the following Preamble and Resolutions were unanimously adopted:

As the relations between the Medical Profession and the Community at large imply reciprocity of duties and obligations, no serious injury can be inflicted on the one by the other without reasonable ground of complaint, and call for amendment by the injured party. If it be for the best interests of the community to have in its midst a body of well-educated and competent physicians, ready for all emergencies of individual disease and wide-spreading pestilence, it is the duty of said community to give encouragement and facilities for physicians to reach, and maintain themselves at, the highest standard of medical attainments and medical ethics, for in this way will they be most serviceable and most efficient. An opposite course, as by the election to offices of trust and emolument of individuals, notoriously false to the standard of medical knowledge and of medical ethics, can only be followed by discouragement, and eventually by the introduction into the community of an inferior body of physicians, who will be found substituting their own random modes of practice and selfish empiricism for the accumulated experience of thousands of years, carefully digested and collated, and wisely and conscientiously applied to the mitigation and cure of disease in all its forms. The wound thus inflicted on the Medical Profession, cannot fail to affect injuriously the community, by depriving it, in the hour of danger and of panic, of a body of zealous and trained men—a health-guard, prepared to repel approaching pestilence, or, if it comes, to abate the violence of its attacks, and to save the lives of their fellow-citizens, often at the sacrifice of their own. If recreants to their first and subsequent pledges, deserters from the ranks of legitimate medicine, and impostors, without even the plea in their favor of lengthened experience, be appointed to offices of trust and emolument, one great incentive is wanting for the regular physician continuing to be true to his Code of Ethics, to keep his plighted faith to his fellow-members of the profession, and to be ready, at any moment, to bring his skill and knowledge into the service of a community which would thus discredit him in so marked a manner, as far, at least, as its public and official action can be exerted. But, despite of all these discouragements and insults, the Medical Profession, in its collective capacity, so long as it is governed by a recognized Code of Ethics, so long as its aspirations are to do the greatest good, just so long is it bound, by imperative duty to itself, and to the community of which it forms a part, not only to repudiate all deserters and breakers of their pledges, but also to disclaim any harmony of sentiment or action with those who, in contravention of their ethical obligations, and as members of a Medical Association, have given, or shall hereafter give, countenance and support to such deserters, either by direct recommendation or by holding professional intercourse with them.

Moved by considerations of this nature, and under the settled convictions of the professional lives of its members, the Philadelphia County Medical Society hereby declares its deep mortification and disgust at the recent appointment to the office of Chief Resident Physician of the Blockley Hospital, of a Dr. James McClintock, a man whose antecedents, in no sense, entitle him to such a mark of public confidence.

A few years ago he proclaimed himself to be a deserter from the ranks of the Profession, by selling whatever reputation he had at the time, together with a certain number of recipes of so-called "Family Medicines," to which his name was attached, but the composition of which was kept secret. These were advertised and recommended in the manner and style usual in the case of other nostrums and quack medicines. Such proceedings are strictly prohibited in the Code of Medical Ethics adopted by the National Medical Association, to the support of whose laws and regulations he, in common with every member of that body, had pledged himself. His expulsion from the Association followed this breach of his pledge and infraction of its laws. The Code of Medical Ethics declares it to be derogatory to professional character "for a physician to hold a patent for any surgical instrument, or medicine, or to dispense a secret nostrum, whether it be the composition or exclusive property of himself or of others. For, if such nostrums be of real efficacy, any concealment regarding it is inconsistent with beneficence and professional liberality; and if mystery alone gives it value and importance, such craft implies most disgraceful ignorance, or fraudulent avarice. It is also reprehensible for physicians to give certificates attesting the efficacy of patent or secret medicines, or in any way to promote the use of them." Every physician, acquainted with the first principles of medicine, knows that the promise held out by the maker and vendor of a quack medicine or nostrum, that it will cure a number of diseases of totally different natures, occurring in both sexes and in all ages, or that it will cure any one disease in all its stages, is, of necessity, false, cruelly false and mischievous, both in its direct consequences in the aggravation of disease, and too often destruction of life, if the article or compound has any activity; and indirectly, by excluding a rational treatment. On these grounds, which are those of common humanity and fair-dealing, quackery is always abhorrent to the mind of every well-educated and conscientious physician.

A few years' perseverance in his evil course of quackery, brought with it disappointment and failure, loss of money, in addition to loss of reputation, and the expelled member having become an applicant for the highly responsible and lucrative office which he now holds, makes an implied acknowledgment of the failure of his speculation on public credulity, by publishing the recipes of his "Family Medicines;" but he makes no formal recantation of previous wrong-doing, or promise of future amendment. There is no assurance offered that he will not engage in similar speculations on a somewhat different foundation. Is such a moment a fitting one for electing this person to an office of trust and emolument, when he is still degraded in the profession; and without waiting for the only legitimate proof of his repentance being regarded as sincere, viz., his restoration to membership in the National Medical Association? In the Philadelphia County Medical Society he has never obtained a footing.

Believing that it fully expresses the sentiments and opinions of not only its own members, but of the Medical Profession throughout the United States, the Philadelphia County Medical Society hereby adopts unanimously the following resolutions:

1st. That it offers its cordial congratulations and assurance of continued interest in their future welfare to the six gentlemen, viz.: Doctors Tutt, of Va.; J. H. Berrien, of Ga.; J. Cummisky, of Phila.; Thomas Marshall, of Va.; X. X. Xaupi, of Missouri, and J. B. Coleman, of Ga., who, at a great sacrifice of opportunities for acquiring clinical experience, resigned, at once, their posts, as Assistant Resident Physicians of the Blockley Hospital, rather than be exposed to the professional intercourse with the obnoxious Chief Resident Physician who had just been appointed.

2d. That any countenance which has been given by a member or members of the Medical Profession, in the way of verbal or written recommendations to the individual who is now Chief Resident Physician of the Blockley Hospital, and an expelled member of the National Medical Association, renders them amenable to censure for a breach of medical ethics, and to the same penalty which has been incurred by this person, with whom they may be said virtually to be in league.

3d. That the prompt resignation of the gentlemen who comprised the consulting medical, surgical, and obstetric staff, at the Blockley Hospital, in order to avoid the stain of official association with the Chief Resident Physician, and in accordance with their known principles and practice, meets with the entire approval of this Society.

4th. That this Society cannot but deprecate the very idea of any of its members holding office under or with the present Chief Physician of the Blockley Hospital, and can only regard such an act as a voluntary loss of membership, and in the case of a physician who is not now a member, as an absolute disqualification for his becoming one.

---

THE LATE MEETING OF THE AMERICAN MEDICAL ASSOCIATION AT NASHVILLE.—The meeting of this body in

May last at the capital of Tennessee, presents several points for comment. In the first place, we had hoped, after what had been said by our esteemed cotemporary the Nashville Journal of Medicine and Surgery, that the medical profession of Nashville would have declined to enter the arena for the office of President of the Association, on the ground that the practice of conferring that post upon a physician, resident at the place where the meeting is held, ought to be abolished. The reasons urged by the Nashville Journal and several similar publications were cogent and full of point, and yet, notwithstanding this, one of the very editors of that periodical allowed himself to be nominated for the office. We mean no disrespect to the distinguished gentleman, when we say that we regret this step, because many of the opponents of the custom, which would certainly be more honored in the breach than in the observance, had reason to believe that it would have been abandoned. It is no honor to a man to be elected to office under such circumstances. It is a concession to the place; or in other words, to good eating and good cheer. We believe there is no other body of a similar kind in this country, among whom a similar custom prevails. The Moderator of the General Assembly of the Presbyterian Church, is seldom, if ever, selected from the clergy of the place where its meetings are held.

Secondly, The American Medical Profession and the American people at large, had a right to expect, after the adoption, by an almost unanimous vote, of the resolution at St. Louis, in 1854, that the custom which, up to that period, had existed, of entertaining the profession with an expensive feast, would not have been revived at the capital of Tennessee. We are told, it is true, that the Profession had no participation in the expense of the ball and supper, but that they were gotten up and paid for by the citizens of Nashville. Would it have been wrong in the Profession of Nashville, to have said to their fellow-citizens, that there was a resolution upon the minutes of the proceedings of the Association, declaring that all public displays of this kind, should henceforth form no part of the *transactions* of that body?

Thirdly, The meeting was a meagre one, both in respect to the number of its attendants, and in the value and importance of its transactions. Hardly any reports were presented; in almost every instance the chairmen of the different committees asked for further time. This is not as it should be. The Association, to carry out the right principles, with which it originally set out, should be more of a working, and less of a pleasure-seeking body. The committees should make it their duty to discharge, with promptness and fidelity, the trust confided to them. It is a bad example to hear so many of them say, that they are "not ready to report;" an example which is apt to become infectious, and to lead ultimately to the past prejudicial results. We should wake up upon this subject. To make the Association what it should be we must work more, talk less, and use a lighter diet. Moreover, the meetings should extend at least over one entire week. It is folly to think that any important business can be transacted in two or three days, by a body so large and unwieldy as this. Besides, the thing is absolutely undignified. As the matter now stands, we literally meet only to adjourn.

---

JEFFERSON MEDICAL COLLEGE.—The chair of Materia Medica and Therapeutics in this Institution, rendered vacant by the resignation of Professor Huston, on account of ill health, has been filled by the appointment of Professor Thomas D. Mitchell, well known as an accomplished lecturer and writer on Materia Medica and Therapeutics. We are happy to add, as will be seen by the following resolutions of the Board of Trustees, that Professor Huston retains his connection with the College as Emeritus-Professor.

*Resolutions of the Board of Trustees of Jefferson Medical College of Philadelphia, on the occasion of the resignation of Professor R. M. Huston.*

*Resolved*, That the Board of Trustees of Jefferson Medical College, in accepting the resignation, by Professor Huston, of the Chair of *Materia Medica and General Therapeutics*, do express their regret at the causes which have given occasion to it, and their fervent hope, that he may yet enjoy many years of restored health and usefulness.

*Resolved*, That the Board, deeply sensible of the benefit which has accrued to the Institution from the talent and zeal so energetically exerted by Professor Huston during the eighteen years in which he has been connected with the Institution, do confer upon him the honorary distinction of *Emeritus Professor of Materia-Medica and General Therapeutics* in the Jefferson Medical College of Philadelphia.

The following Resolutions were unanimously adopted by the Faculty, upon learning that Professor Huston had vacated his chair.

*Resolved*, That whilst the Faculty of Jefferson Medical College have had the apprehension for some time, that the impaired health of Professor Huston might lead him to resign his position as Professor of Materia Medica and General Therapeutics, they had indulged a hope, that he might still be able to execute the duties devolving upon him in a manner that might be creditable to himself, and advantageous to the Institution.

*Resolved*, That the Faculty do deeply deplore the circumstances which have led to the resignation of one who has so zealously and efficiently labored in the great cause, in which they have all been engaged so long, and so harmoniously, and with a success unexampled in the history of similar Institutions.

*Resolved*, That the Dean of the Faculty do communicate these resolutions to Professor Huston, with an assurance, that the most respectful and affectionate regards are entertained for him by his colleagues ; and that he has their profound sympathy in his sufferings, and their sincere wishes for his entire restoration.

---

CHANGES IN MEDICAL SCHOOLS.—Dr. Theodore S. Bell, of Louisville, has been appointed to the chair of Medicine in the University of Louisville, in the place of Professor Rogers, who will hereafter lecture exclusively on Clinical Medicine. Dr. George W. Bayless, formerly Demonstrator of Anatomy in the University of Louisville, has received the appointment of Professor of Physiology and Pathological Anatomy in the Kentucky School of Medicine. The Miami Medical College at Cincinnati has been absorbed by the Medical College of Ohio, which takes four of its professors, Judkins, Comegys, Foote, and Mendenhall, leaving all the others unprovided for, excepting Dr. Armor, who has just received the appointment of

Professor of Pathology and Clinical Medicine in the Missouri Medical College of St. Louis. Dr. George S. Blackie, a graduate in medicine of the University of Edinburgh, has been elected to the chair of Botany in the Collegiate Department of the University of Nashville. Dr. J. C. Nott, of Mobile, takes the chair of Anatomy in the University of Louisiana, rendered vacant by the resignation of Professor Wederstrandt.

---

FOREIGN TEXT-BOOKS.—The St. Louis University, in its Annual Announcement for the current year, recommends twenty-five text-books, of which only three are American! We take it for granted, that the Institution, which furnished the American Medical Association with its President (at its meeting three years ago), has not a very exalted appreciation of American authors.

In the Jefferson Medical College, of fifteen text-books, mentioned in the Annual Announcement, four and a *half* are foreign, the remainder being the works of its own professors, with the exception of the excellent treatise on the Diseases of Children, by the younger Meigs. The *half* book is the work of our learned collaborator (and an excellent one it is), Dr. John Bell, as comprised in Bell and Stokes's Practice of Medicine. Professor Pancoast recommends Wilson's Anatomy, and his edition of Quain's Anatomical Plates. Professor Bache seems to prefer Fownes's Chemistry to that of Draper or Silliman.

The Pennsylvania Medical College enumerates thirty-five text-books, of which ten are American; of the latter, four are works of its own Faculty.

Of the number and nature of the text-books used in the University of Pennsylvania, we are unable to give any account, as no mention of them is made in the Annual Announcement of the School.

The Philadelphia College of Medicine recommends thirty-one text-books, of which nine are American.

---

OPHTHALMOLOGICAL CONGRESS.—Physicians and Surgeons, in all parts of the world, interested in the study of Ophthalmology, are invited to convene at Brussels, on the 13th of September next, to deliberate upon the best method of arresting the spread of inflammation of the eye, so common in some of the continental armies of Europe. It is stated, in the circular calling attention to the subject, that the disease is of frightful extent and virulence, that it has already desolated several armies, and that it counts daily new victims among the people with whom the disabled soldiery come in contact, thus clearly showing its contagious character. Statistics of this disease, and other matters of ophthalmic interest, will engage the attention of the meeting. Invitations have been issued to different scientific physicians and surgeons in the United States. We regret to learn that there will be no delegates from this city, although we understand that interesting communications have been forwarded by Dr. Hayes, Dr. Littell, and Professor Gross.

---

CONGRESS OF GERMAN PHYSICIANS AND NATURALISTS.—This distinguished body will convene at Bonn, on the 18th September, and be in session one week.

---

MARRIAGES OF CONSANGUINITY.—Dr. Bemiss, of Louisville, Kentucky, whose article on "Marriages of Consanguinity," in a former number of the Review, has been so extensively copied by European journalists, proposes to continue his inquiries into the subject, and with this view invites contributions from his professional brethren, in all parts of the country, on the following points, to be forwarded to him by the 18th of January, 1858.

1. The degree of Consanguinity of parties (whether first, second, third, or fourth cousins; or, as in rare instances, uncle and niece).

2. The date (approximative) of marriage.

3. The number, sex, and *condition* of children born to each marriage.

4. The number of children dead, cause of death, and age when known.

5. The constitution, temperament, and occupation of parents, with any habits or circumstances calculated either to favor or retard the normal development of offspring.

---

FOREIGN HONORS TO AN AMERICAN SURGEON.—Dr. W. J. Holt, of Augusta, Ga., has just received, through the Russian minister to this country, the "decoration" of Commander of the Imperial Order of St. Stanislaus, in consideration of his services during the campaign in the Crimea. The cross is of massive gold, and beautifully wrought. Dr. Holt was appointed member of the Order of St. Anne, while still in the service of Russia; and this second compliment, now that he has left that service, testifies to the Czar's appreciation of the ability with which the surgeon's duties were discharged.

---

NEW MEDICAL WORKS.—Messrs. Lippincott & Co. have in press, and will issue, early in October, a revised and enlarged edition of Professor Thomas D. Mitchell's Materia Medica and Therapeutics. The same firm will also publish, at an early day, a treatise, by the same author, on Fevers, with special reference to these diseases as they prevail in the United States. The work will form an octavo volume of about 650 pages, and will comprise a full and comprehensive digest of the existing state of the science, with an incorporation of the author's personal experience.

---

Professor Austin Flint, of Buffalo, the former able and accomplished lecturer of medicine in the University of Louisville, is actively engaged upon the preparation of a work on the Diseases of the Heart, a subject which has, for many years, occupied much of his attention at the bedside, both in private and hospital practice. To those who are acquainted with Professor Flint's writings, it is needless to say that he will produce a work which will be alike creditable to himself and honorable to our national medical literature.

---

NUMBER OF STUDENTS AT THE GERMAN UNIVERSITIES.—The *Moniteur des Cours Publiques* gives the following as the number of students at the German Universities in 1856, with the exception of Vienna:

| | Germans. | Foreigners. |
|---|---|---|
| Berlin, | 211 | 54 |
| Bonn, | 90 | 6 |
| Breslau, | 128 | 13 |
| Erlangen, | 102 | 4 |
| Fribourg, | 44 | 4 |
| Giessen, | 121 | 25 |
| Göttingen, | 77 | 78 |
| Geisswald, | 99 | 2 |
| Halle, | 47 | — |
| Heidelberg, | 60 | 61 |
| Jena, | 38 | 11 |

| | Germans. | Foreigners. |
|---|---|---|
| Kiel, | 38 | 4 |
| Konigsberg, | 81 | 4 |
| Leipsic, | 167 | 59 |
| Marburg, | 65 | 8 |
| Munich, | 202 | 37 |
| Rostock, | 19 | — |
| Tubingen, | 100 | 11 |
| Wurzburg, | 97 | 222 |

ELECTION.—We have just learned that Dr. Wm. H. Gobrecht, of this city, has been elected to the chair of Anatomy, in the Philadelphia College of Medicine.

NECROLOGICAL NOTICES.—Lately, at Lacken, Sir Robert Carswell, Physician in Ordinary to the King of the Belgians. The deceased was born at Thornbank, in Scotland, and had attained his 64th year. He was one of the largest contributors to the "Encyclopædia of Practical Medicine," and was the author of "Illustrations of Morbid Anatomy," a work which is well-known to the profession of this country.

Sir James Eyre, M.D., L.R.C.P., died suddenly on June 19th. He was the author of "The Stomach and its Difficulties."

"Sir James Eyre was born in 1792. His father was a Buckinghamshire clergyman. From 1806 to 1811 he was a pupil of Mr. Devanport, a Surgeon of Harborough. In 1811 he became a house pupil of Mr. Phillips, the Surgeon to the Marylebone Infirmary. In 1812, he became House Surgeon to this Infirmary. He attended the Windmill Street School, and St. Bartholomew's, and in 1813 went to Hereford to assist the late Mr. Griffiths. He came to town in 1814 to pass the College of Surgeons, and returned to Hereford to practise as a Surgeon. He married in 1816, worked hard as a surgeon, was elected a member of the corporation, and in 1829 Mayor of Hereford; his father, then 78 years of age, coming to preach the inauguration sermon. In 1831, on the accession of William IV, an address of congratulation was presented to the sailor king, and the Mayor of Hereford was knighted, the Mayors of Hereford and Liverpool being the only two who received the distinction on that occasion. From 1831 to 1833 Sir James studied medicine afresh in Edinburgh and Paris, and took the degree of M.D. at Edinburgh, in 1834. He settled in London in the same year, and was elected Physician-Accoucheur to the St. George's and St. James's Dispensary, which office he filled for seventeen years, when he gave up midwifery practice. He became a licentiate of the College of Physicians in 1836. In 1845 he published his work on "Exhausting Diseases," strongly recommending the oxide of silver. In 1852 his "Stomach and its Difficulties" ap-appeared. For several years past he resided in Brook Street, but he never had an extensive medical practice in London. He attended Her Majesty's *levée* on Thursday, and was quite well until late that evening, but on Friday morning he had gone from among us."—*London Med. Times and Gazette.*

Died, May 28th, at Palermo, in Sicily, in the 80th year of his age, John Howell, M.D., Deputy Inspector-General of Military Hospitals.

